# Causes of preterm and low birth weight neonatal mortality in a rural community in Kenya: evidence from verbal and social autopsy

**DOI:** 10.1186/s12884-021-04012-z

**Published:** 2021-07-29

**Authors:** Beatrice Olack, Nicole Santos, Mary Inziani, Vincent Moshi, Polycarp Oyoo, Grace Nalwa, Linet Christopher OumaOtare, Dilys Walker, Phelgona A. Otieno

**Affiliations:** 1 Centre for Clinical Research, Kenya Medical Research Institute, P.O Box 54840 00200, Nairobi, Kenya; 2 University of California San Francisco, Institute for Global Health Sciences, San Francisco, California, USA; 3 Department of Paediatrics, School of Medicine, Maseno University, P.O Box Private Bag, Maseno, Kenya

**Keywords:** Preterm birth, Verbal autopsy, Neonatal mortality, Social autopsy, Kenya

## Abstract

**Background:**

Under-five mortality in Kenya has declined over the past two decades. However, the reduction in the neonatal mortality rate has remained stagnant. In a country with weak civil registration and vital statistics systems, there is an evident gap in documentation of mortality and its causes among low birth weight (LBW) and preterm neonates. We aimed to establish causes of neonatal LBW and preterm mortality in Migori County, among participants of the PTBI-K (Preterm Birth Initiative-Kenya) study.

**Methods:**

Verbal and social autopsy (VASA) interviews were conducted with caregivers of deceased LBW and preterm neonates delivered within selected 17 health facilities in Migori County, Kenya. The probable cause of death was assigned using the WHO International Classification of Diseases (ICD-10).

**Results:**

Between January 2017 to December 2018, 3175 babies were born preterm or LBW, and

164 (5.1%) died in the first 28 days of life. VASA was conducted among 88 (53.7%) of the neonatal deaths. Almost half (38, 43.2%) of the deaths occurred within the first 24 h of life. Birth asphyxia (45.5%), neonatal sepsis (26.1%), respiratory distress syndrome (12.5%) and hypothermia (11.0%) were the leading causes of death. In the early neonatal period, majority (54.3%) of the neonates succumbed to asphyxia while in the late neonatal period majority (66.7%) succumbed to sepsis. Delay in seeking medical care was reported for 4 (5.8%) of the neonatal deaths.

**Conclusion:**

Deaths among LBW and preterm neonates occur early in life due to preventable causes. This calls for enhanced implementation of existing facility-based intrapartum and immediate postpartum care interventions, targeting asphyxia, sepsis, respiratory distress syndrome and hypothermia.

**Supplementary Information:**

The online version contains supplementary material available at 10.1186/s12884-021-04012-z.

## Background

The World Health Organization (WHO) estimates that 15 million babies are born preterm annually and approximately one million succumb to death in their first 4 weeks of life due to complications of prematurity[1]. Preterm birth has been reported to be the leading cause of neonatal death [1–5]. Infants born preterm are four times more likely than term infants to die during the neonatal period (first 28 days) and infancy (first year) [6]. Mortality rates increase proportionally with decreasing gestational age or birth weight [7, 8]. Preterm babies born in developed countries have almost ten times better survival rates compared to those born in low-resource settings ([4] http://www.who.int/maternal_child_adolescent/documents/born_too_soon/en/). South Asia and sub-Saharan Africa account for over three-quarters of the world’s newborn deaths due to preterm birth and its complications [9]. Most deaths in preterm babies in the developing nations occur from preventable causes such as infection, asphyxia, hypothermia, and hypoglycemia. Moreover, > 80% are born between 32 and 37 weeks of gestation (moderate/late preterm), and many die needlessly for lack of simple, essential care such as warmth and feeding support [10].

Babies born weighing less than 2500 g are termed low birth weight. Their births can be related to premature delivery, as well as, associated with the restriction of intrauterine growth or the relationship between both situations [11]. In sub-Saharan Africa, 14 percent of babies are born with low birthweight (LBW), and have an infant mortality rate 77 times higher than normal-weight babies [11].

The Every Newborn Action Plan (ENAP), a road map to reduce preventable neonatal deaths advocates for strengthening death surveillance through supporting the community in reporting and reviewing causes of maternal and neonatal deaths [12, 13]. Kenya ranks sixth among the African countries with the most newborn deaths [14]. The 2014 Kenyan demographic health survey documents that five years preceding the survey under-five mortality rate declined from 74/1000 to 52/1,000 live births and infant mortality decreased from 52/1,000 to 39/1,000 live births however, neonatal mortality rates declined from 31/1,000 to only 22/1,000 live births [15]. Despite the progressive decline in under five and infant mortality, there has been slow progress in reducing neonatal mortality.

Understanding causes of and circumstances preceding both preterm and low birth weight neonatal death, is essential for accelerating progress towards Sustainable Development Goal (SDG) 3, target 3.2, that aims at reducing neonatal mortality rates to 12 per 1,000 live births by 2030. Integrating verbal and social autopsy has given compelling results for improving maternal and child survival estimates. In Burkina Faso and Indonesia, D’Ambruoso et al. demonstrated that VASA results can be used for planning and prioritization of interventions that improve maternal health [16]. In emergencies, VASA has been used to identify gaps in civil registration and strengthen health systems in times of crisis. In Niger, the use of VASA data with effective feedback led to evidence-based decision-making and program improvement in child health [3, 15–18].

Effective and efficient Civil Registration and Vital Statistics (CRVS) is the gold standard for generating reliable data on demography, mortality, and causes of death data [19], however, it is estimated that more than half the deaths that occur among Kenyan neonates remain unreported [20].

This study aimed to determine the causes of death among preterm and low birth weight (LBW) neonates born in selected health facilities in Migori county between January 1, 2017, to December 31, 2018.

## Methods

### Study design

We conducted a descriptive cross-sectional study for all preterm and low birth weight (LBW) neonatal deaths that occurred January 1, 2017, to December 31, 2018 in 17 selected health facilities in Migori County, Western Kenya.

### Study population

The study population included a cohort of preterm and LBW neonates enrolled in an implementation science study conducted by the Preterm Birth Initiative (PTBi), a collaboration among Kenya Medical Research Institute (KEMRI), Makerere University and University of California, San Francisco. The initiative employed a package of selected interventions to improve birth outcomes and reduce morbidity and mortality of preterm and low birth weight babies in selected health facilities in Migori County, Kenya and Busoga region in Uganda. The study areas and the intervention package are described in detail in the published study protocol [21].

In Kenya, the 17 selected health facilities in Migori County, included one county referral hospital, 14 government health facilities and two missionary hospitals. The selected facilities were high volume in terms of annual deliveries compared to other facilities within the county. All babies born with birth weight < 2500 g (LBW); or birth weight ≥ 2500 and < 3000 g with documented or calculated gestational age less than 37 weeks (preterm) were eligible for enrollment into the study. Upon consenting, mothers who delivered live low birth weight and preterm babies were followed up to determine status of the baby at day 28. The baby’s status was recorded in the PTBi database.

The caregivers of babies who had died within 28 days of life were invited to participate in the VASA study. Babies who died before discharge were identified from the health facility maternity registers and the ones who died post-discharge were identified from the PTBi database. We abstracted identifying contact information of the deceased neonates onto the VASA study locator form. The provided phone contact and/or physical location information was used to reach out to the mothers/caregivers of the deceased neonates for an appointment. In case the contact information was missing, or the provided contact was unreachable after three phone attempts, Community Health Volunteers helped to trace the study participant within the indicated village of residence. A caregiver was declared lost to follow up after 3 attempts using all possible methods to trace her.

### Data collection

The identified households were visited by research assistants trained on VASA to administer the VASA questionnaire for data collection. This was done at least two weeks after the death of the baby, to allow for the mourning period. The appropriate respondent was the person involved in primary care for the neonate before he/she died. In most cases this would be the mother, however, secondary respondents were allowed, if necessary, to capture information on all phases of the illness, including the mother’s pregnancy and delivery, during which she may herself have been too ill and unaware of the neonates’ condition. For respondents who had multiple neonatal deaths, the questionnaire was administered for each baby except for the socio-demographic section.

The VASA questionnaire used for data collection was adapted from the WHO standardized verbal autopsy questionnaire for deaths that occur before 28 days [22] and social autopsy questionnaire from the Child Health Epidemiology Reference Group (CHERG) [23]. The questionnaire is divided into three main sections; the first section covers general information for deceased neonates, demographics of the deceased, and household and socio-demographic characteristics of the respondent. The second section covers the circumstances surrounding the child’s death, including signs and symptoms of any illness, the caregiver’s perception of the illness, actions taken and care sought. Any barriers to seeking care are also noted. In addition to neonatal deaths, this section also asks questions about the maternal history, including the mother’s antenatal care and care-seeking for obstetric complications, and about newborn care before and during the illness. The third section is an open narrative that allows the caregiver to narrate about the baby’s illness and events preceding death in his or her own words. Any health records provided by the caregiver describing the treatment the child received were also noted.

### Assigning cause of death

To assign a probable cause of death, two clinicians trained on the WHO International Classification of Diseases (ICD 10) independently reviewed the signs and symptoms recorded on the questionnaires. If the same diagnosis was reached by the two physicians, this was accepted as the probable cause of death. If there was a discrepancy between diagnoses, an independent third physician was involved to determine a consensus on the probable cause of death.

### Data management and analysis

Completed questionnaires were checked for completeness, validity and reliability. Data were entered into a Microsoft Access database then transferred into Stata 12 statistical software for cleaning and analysis. Descriptive statistics presented measures of central tendencies for quantitative data, including mean (standard deviation), median (range) and frequency distributions (frequencies and percentages). Data were presented in tables and graphs.

Reporting of the study conforms to the Strengthening the Reporting of Observational Studies in Epidemiology (STROBE) statement [24].

## Results 

**Fig. 1:**
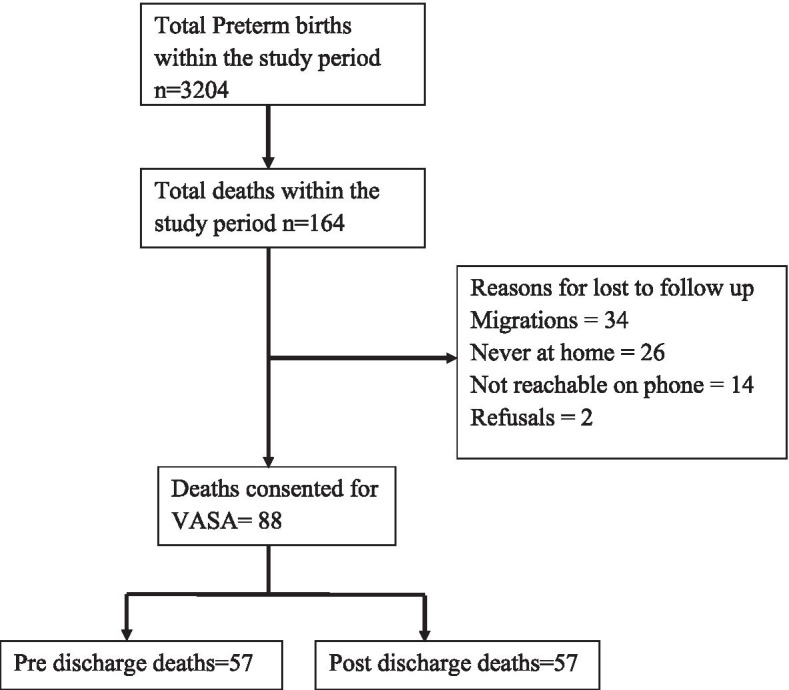
Flow of the Study Participants

During the VASA study period (January 1, 2017, to December 31, 2018, 3204 (8.0%) babies were born preterm or LBW and a total of 164 (5.1%) of the neonates died during the study period. Among the neonates who died, we conducted VASA for 88 (53.7%) deaths. Figure 1 illustrates the flow of the study population.

Socio-demographic, household and obstetric characteristics of mothers and characteristics of the deceased neonates are shown in Table 1. A total of 82 mothers were interviewed, among them were six mothers who had multiple births. The majority of the mothers 74 (91.4%) were above 18 years and all had some formal education. Farming was the mother’s main occupation. The distribution of male and female neonates who died was similar. Over three-quarters of the neonates died in the health facilities within their first week of life. Breast milk feeding within the first hour was initiated among 53/84 (63%) neonates while kangaroo care was practiced on only one neonate.

**Table 1:** Characteristics of mothers and deceased neonates

**Mothers of deceased neonates (*n* = 82)**	
**Age in Years**	n (%)
< 18	7 (8.5)
18 ‒ < 35	71 (86.6)
≥ 35	4 (4.9)
**Maternal Education**	
Primary	34 (41.5)
Secondary	40 (48.8)
Tertiary +	8 (9.9)
**Mothers Occupation**	
Farmer	31 (37.8)
Unemployed	25 (30.5)
Employed	6 (7.3)
Casual labourer	20 (24.4)
**Source of cooking energy**	
Firewood	51 (62.2)
Charcoal	25 (30.5)
Other	6 (7.3)
**Multiple gestations**	
Yes	6 (7.3%)
No	76 (92.7%)
**Mode of delivery**	
Spontaneous vertex delivery	57 (69.5)
Caesarean section	12 (14.6)
Breech delivery	11 (13.4)
Assisted vaginal delivery	2 (2.4)
**Characteristic of deceased neonates *n* = 88**	
Male	40 (45.5)
Female	48 (54.6)
**Gestational age in weeks**	
< 28	20 (22.7)
28 to < 32	19 (21.6)
32 to < 37	32 (36.4)
≥ 37	17 (19.3)
**Weight at birth**	
< 1000	17 (19.3)
1000 to < 1500	27 (30.7)
1500 to < 2000	17 (19.3)
2000 to < 2500	12 (13.6)
≥ 2500	15 (17.1)
**Place of death**	
Health facility	69 (78.4)
Home	12 (13.6)
En route to a health facility	2 (2.3)
Informal health provider	5 (5.7)

### Care pathway for the deceased neonates

**Fig. 2:**
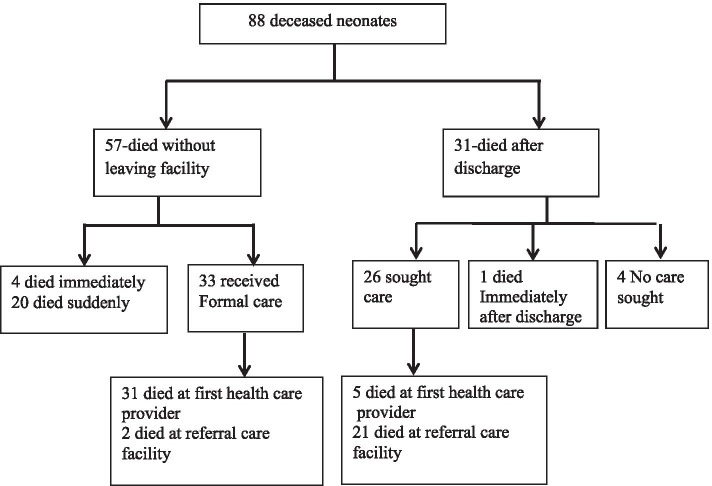
Pathway to seeking care for 88 neonatal deaths

Mothers of 84 neonates who died recognized some signs and symptoms of the baby’s illness. The symptoms commonly reported were difficulty in breathing (59.1%), fast breathing (12.5%) and feeling cold to touch (11.4%) among others listed in Table 2 below. Figure 2 illustrates the care seeking pathway to the deceased neonates. Four babies died immediately after birth while 20 babies died suddenly at the health facility before discharge. Formal care defined as care by a formally trained and certified health care practitioner, was administered to 33/57 (57.9%) babies at the health facility before discharge. Among thirty one babies who had been previously discharged from the health facility to go home, formal care was sought for 26/31 (83.9%) babies and majority of them 21/26 (80.8%) were referred to a second health care provider. One mother did not take the baby for formal healthcare citing that it was late at night and there was no available means of transport to the hospital. Mothers of three neonates who did not seek any care for their babies did not perceive the illness to be severe as to warrant immediate medical attention.

**Table 2:** Symptoms neonates suffered before death

*Symptoms	n (%)
Difficulty in breathing	52 (59.1%)
Fast breathing	11(12.5%)
Felt cold to touch	10 (11.4%)
Grunting	6 (6.8%)
Fever	5 (5.7%)
Chest In drawing	4 (4.5%)
Vomiting	2 (2.3%)
Wheezing	1 (1.1%)

^*^multiple symptoms reported

### Distribution of causes of death among preterm or LBW neonates

**Fig. 3:**
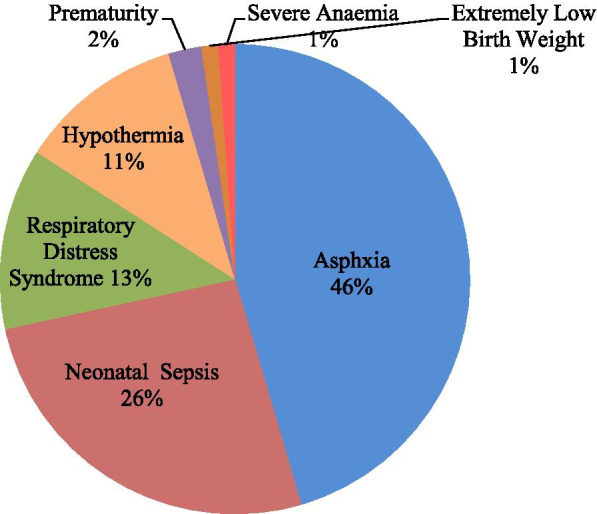
Causes of death among preterm and low birth weight neonates

Figure 3 below illustrates the distribution of causes of death by ICD 10 coding. Birth asphyxia (45.5%), neonatal sepsis (26.1%) and respiratory distress syndrome (12.5%) were the three leading causes of neonatal deaths.

### Distribution of causes of death by discharge status and neonatal period

Asphyxia, respiratory distress syndrome, and hypothermia, were the leading causes of death for babies who died before discharge. Neonatal sepsis was the leading cause of death for babies who were discharged from the health facility. In the early neonatal period (0–6 days) the leading causes of death were asphyxia (54.3%), sepsis (15.7%), respiratory distress syndrome (15.7%), and hypothermia (10.0%). Among late neonatal deaths (7–28 days), sepsis (66.7%), hypothermia (16.7%) and asphyxia (11.1%) were the leading causes. See Table 3 below.

**Table 3:** Causes of death by discharge status and neonatal period

Causes of death	Discharge status		Neonatal period	
	**Pre-discharge** ***n* = 57**	**Post-discharge** ***n* = 31**	**Early (0-6 days)** ***n* = 70**	**Late (7–28 days)** ***n* = 18**
Asphyxia	33 (57.9)	7 (22.6)	38 (54.3)	2 (11.1)
Sepsis	6 (10.5)	17 (54.8)	11 (15.7)	12 (66.7)
Respiratory distress syndrome	9 (15.8)	2 (6.5)	11 (15.7)	0 (0)
Hypothermia	8 (14.0)	2 (6.5)	7 (10.0)	3 (16.7)
Prematurity	0 (0)	2 (6.5)	2 (2.9)	0 (0)
Extremely low birth weight	1 (1.7)	0 (0)	1 (1.4)	0 (0)
Severe anaemia	0 (0)	1 (3.2)	0 (0)	1 (5.6)

**Fig. 4:**
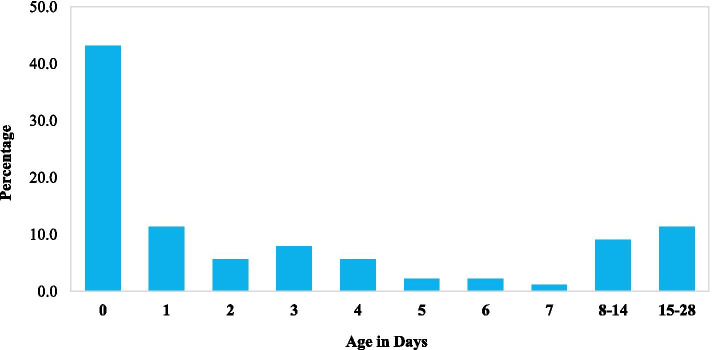
Neonates age at the time of death in days

The distribution of age at the time of death in Fig. 4 below indicates that the proportion of babies who died in the first week was 78.4%. A total of 38 (43.2%) neonates died within the first 24 h and of these, 53% died pre-discharge.

### Death by gestational age

**Fig. 5:**
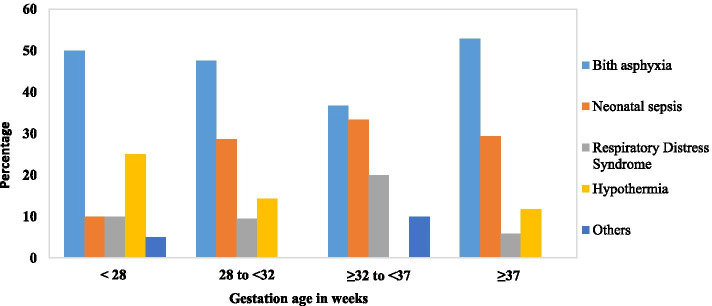
Causes of neonatal deaths stratified by Gestational Age

Among children born less than 37 weeks, the proportion of death due to asphyxia was almost similar across the different gestational age groups. The proportion of death due to neonatal sepsis and respiratory distress syndrome increased with increasing gestational age peaking at > / = 32 to < 37, and declined slightly at > / = 37, while deaths due to hypothermia reduced with increasing age (Fig. 5).

## Discussion

The leading probable causes of the neonatal death among preterm or LBW infants were birth asphyxia (45.5%), neonatal sepsis (26.1%), respiratory distress (12.5%), and hypothermia (11%). These findings reaffirm the report of other studies in Ghana and Bangladesh that indicate that most neonates die as a result of prematurity, birth asphyxia and sepsis [25].

Asphyxia accounted for almost half of all the neonatal deaths. This proportion is comparable to that reported in a rural sub-district in Bangladesh [26]. However, in a recent population-based study in several countries, Kenya reported lower proportions of deaths from birth asphyxia (32%) and almost similar proportions for infections (28%) [27]. A recent study from Ethiopia reported asphyxia as the cause in 14% of the deaths in neonates. The observed difference in higher proportion of deaths due to asphyxia in our study is possibly because of methodology issues. The Kenyan study was conducted among all neonates and not necessarily preterm or low birth weight infants while the Ethiopian study used clinical post mortem examinations to determine the cause of death.

Neonatal sepsis caused almost one-quarter of all deaths, the majority occurring after discharge. These proportions are similar to what is reported in other African countries ranging from 10–26% [28].

The distribution of causes of death in the early neonatal period is similar to results cited in other studies whereby the leading cause of death in early neonatal period is asphyxia while the leading cause of death in the late neonatal period is sepsis [29].

Physical characteristics and environmental factors predispose the preterm infant to hypothermia [30]. In this study, we observed a low prevalence of both skin to skin contact and early initiation of breast milk within the first hour. Lack of these essential new-born practices may have resulted to the deaths due to hypothermia with the impact being higher among children born at lower gestational ages. To avert these deaths from hypothermia, proven high impact yet low-cost interventions of preterm babies, like early initiation to breast milk and practicing kangaroo mother care, is quite essential [31]. A meta-analysis of 15 studies concluded that KMC substantially reduced neonatal mortality among preterm and was highly effective in reducing morbidity [32]. The PTBI East Africa recently documented that quality data, strengthened provider skills and teamwork emphasising on antenatal corticosteroids, immediate skin to skin care, preterm feeding and new-born resuscitation are evidence based practices that decrease neonatal mortality [33].

Preterm and LBW neonates are very vulnerable in the first week. Consistent with other studies, we found that more than three-quarters (78.4%) of the neonatal deaths occurred in their first week of life and approximately half of these deaths occurred in the first 24 h [29]. Thus, there is a need for refocused antenatal care, safe delivery practices and continuous engagement with mothers on the care of the preterm or LBW babies in and beyond the hospital walls.

Majority of mothers were able to recognize signs and symptoms of illness their neonates suffered and sought formal health care for them. In most cases, the neonates suffered multiple symptoms that would have benefitted from integrated management of neonatal and childhood illnesses. The reasons reported by four mothers for not seeking formal health care point to mothers’ perceptions on illness severity and how distance as well as transportation act as barriers to seeking healthcare for the sick neonates. Our findings concur with findings of systematic review conducted in Low and Middle Income countries as well review in Sub Saharan Africa which indicate that individual factors as well as social factors such as transportation and distance to health facility result in severe morbidities and eventually mortality among the neonate [34].

Given that majority of the deaths occurred in the health facilities, this points to the need of strengthening the health systems for the care of the preterm and LBW neonates to avert deaths. The United Nations Inter agency group for child mortality estimation in their report level and trends in child mortality 2020 states that quality care at birth and skilled treatment immediately after birth and in the first days of life averts neonatal deaths from preventable causes of death such as prematurity birth asphyxia, infections and birth defects [35].

### Strength and limitations

We adapted the used of WHO standardized tools for collecting and interpretation of the causes of deaths. The study population was drawn from the county's high volume facilities, where most preterm and low birth weight deliveries occurred.

Self-reported symptoms have limited reliability when reported by lay-persons and can be subjective. We assumed that the cause of death investigated had symptoms that were easily recognized and recalled by the primary caregiver. To reduce recall bias the research assistants conducted the interviews at least two weeks after the death occurred.

The study provided no information for neonatal deaths that occurred among preterm and LBW babies delivered at home or in other non-selected health facilities. Thus, the findings in this study cannot be generalized to the entire population and need to be interpreted with caution due to potential bias associated with lost to follow up. Selection bias occurred due to numbers of neonates lost to follow up. However, the VASA neonates and the lost to follow up neonates had similar baseline characteristics that included maternal age, gestation age and birth weight. The neonates lost to follow up were more likely to be male while VASA neonates experienced more pre discharge deaths. See supplementary table.

## Conclusion

Birth asphyxia, neonatal sepsis, respiratory distress, and hypothermia, were the major causes of deaths of the neonates participating in the study. A big proportion of deaths happened within the first 24 h of life. It is important to note that all these conditions are preventable thus, enhancing low-cost life-saving interventions during the intrapartum care and the immediate neonatal period will go a long way to change this trend. A large well-designed study is recommended to establish risk factors associated with the deaths among the preterm and low birth weight babies.

## Supplementary Information

**Additional file 1: Supplementary Table.** Characteristics of VASA infants versus infants who were lost to follow up.

## Data Availability

The datasets used to support the findings of this study are available from University of California San Francisco upon reasonable request and signing a data access agreement subject to approval from the study principal investigators.

## Abbreviations

CHERG: Child Health Epidemiology Reference Group
CRVS: Civil Registration and Vital Statistics
ENAP: Every Newborn Action Plan
GA: Gestational Age
ICD-10: International Classification of Diseases
LBW: Low Birth Weight
KEMRI: Kenya Medical Research Institute
KMC: Kangaroo Mother Care
PTBI-K: Preterm Birth Initiative-Kenya
SDG: Sustainable Development Goal
SERU: Scientific and Ethical Review Unit
UCSF: University of California, San Francisco
VASA: Verbal and Social Autopsy
WHO: World Health Organization
