# Supplementary material for: Causes of preterm and low birth weight neonatal mortality in a rural community in Kenya: evidence from verbal and social autopsy
Source: BMC Pregnancy Childbirth. 2021 Jul 29;21:536. doi: 10.1186/s12884-021-04012-z (PMC8320164; doi:10.1186/s12884-021-04012-z)
Supplement: Supplementary file 1 — Additional file 1: Supplementary Table. Characteristics of VASA infants versus infants who were lost to follow up. [file 12884_2021_4012_MOESM1_ESM.docx]

**Supplementary Table**

**Characteristics of VASA neonates versus neonates who were lost to follow up**

| **Characteristic** | **VASA neonates**  **(n=88)** | **Lost to follow up neonates (n=76)** | **p value** |
| --- | --- | --- | --- |
| **Maternal Age (years)**  <18  ≥18-35  >35 | 7 (7.9)  76 (86.4)  5 (5.7) | 12 (15.8)  56 (73.7)  8 (10.5) | 0.19 |
| **Baby’s Sex**  Male  Female | 39 (44.3)  49 (55.7) | 47 (61.8)  29 (38.2) | < 0.05 |
| **Baby GA**  < 28  28 to <32  32 to <37  ≥ 37 | 19 (22.7)  19 (21.6)  32 (36.4)  17 (19.3) | 14 (18.7)  21 (28.0)  29 (38.7)  11 (14.7) | 0.67 |
| **Baby Birth Weight**  < 1000  1000 to <1500  1500 to <2000  2000 to <2500  ≥2500 | 17 (19.3)  23 (26.1)  18 (20.5)  14 (15.9)  16 (18.2) | 12 (15.8)  16 (21.1)  24 (31.6)  15 (19.7)  9 (11.8) | 0.39 |
| **Discharge status**  Pre discharge death  Post discharge deaths | 57 (64.8)  31 (35.2) | 37(48.7)  39(51.3) | < 0.05 |
